# Continuous Flow Technology as an Enabler for Innovative Transformations Exploiting Carbenes, Nitrenes, and Benzynes

**DOI:** 10.1021/acs.joc.2c00963

**Published:** 2022-06-14

**Authors:** Kian Donnelly, Marcus Baumann

**Affiliations:** School of Chemistry, Science Centre South, University College Dublin, D04 N2E2 Dublin, Ireland

## Abstract

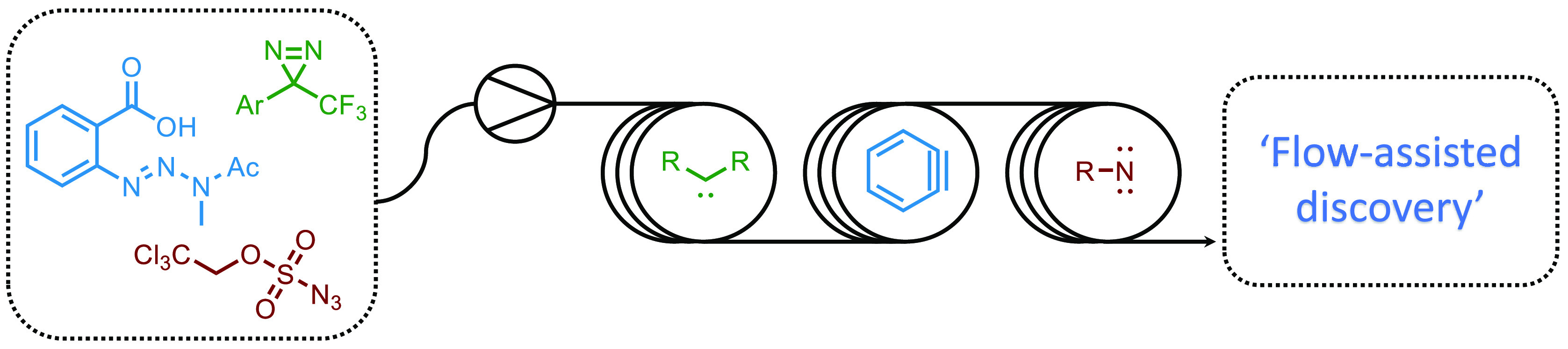

Miniaturization offered by microreactors provides for superb reaction control as well as excellent heat and mass transfer. By performing chemical reactions in microreactors or tubular systems under continuous flow conditions, increased safety can be harnessed which allows exploitation of these technologies for the generation and immediate consumption of high-energy intermediates. This Synopsis demonstrates the use of flow technology to effectively exploit benzynes, carbenes, and nitrenes in synthetic chemistry programs.

The advantages of carrying out reactions in continuous flow are well documented.^1−4^ Characteristics such as improved heat and mass transfer are particularly useful for reactions which involve unstable intermediates, as they provide superior reaction control compared to their batch counterpart. This allows for a potential expansion of chemical space, accessing reactions that were not previously possible due to intermediates being deemed too reactive. The development of commercially available flow reactors has increased its uptake as an enabling technology. This has resulted in the field rapidly advancing over the past decade, in particular its use in the generation of reactive intermediates. Extensive and informative reviews of a range of reactive intermediates such as diazo compounds, ketenes, and highly reactive organometallic species have been published in recent years.^1,4−7^ This Synopsis aims to highlight recent reports of continuous flow technology as an enabler for the exploitation of carbenes, nitrenes, and benzynes as highly reactive intermediates.

## 1 Generation and Trapping of Arynes

Since the development of a mild method for the generation of benzynes by Kobayashi in 1983, the synthetic utility of arynes has been widely investigated.^8−10^ This method generates benzyne from trimethylsilylaryl triflates and a fluoride source.^11^ The unstable benzyne intermediate can then react with a range of substrates, with many examples published in the literature. Arynes have found use in the step-economic synthesis of various heterocycles.^8,9^

Reactions utilizing arynes typically involve the formation of a range of side products, which in some cases can be avoided through careful control of reaction parameters. Carrying out reactions in continuous flow provides this through improved mass and heat transfer, which prevents local temperature hot spots which are sometimes observed when reactions are carried out in batch mode.

**Scheme 1:**
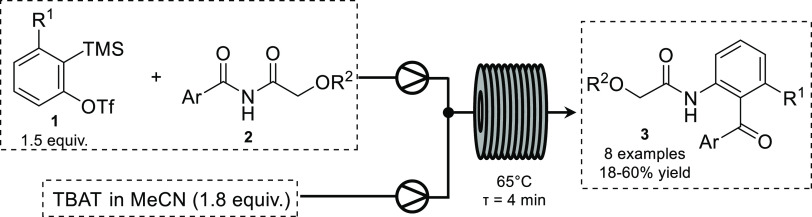
Continuous Flow Aryne–Imide Insertion

An issue commonly encountered when transferring a reaction from batch to continuous flow mode is the solubility of reagents. The generation of arynes via Kobayashi’s method requires the presence of a fluoride source, typically CsF; however, due to its limited solubility in organic solvents, alternatives are required in continuous flow. In 2016, Khadra and Organ reported the *in situ* generation and subsequent Diels–Alder reaction of benzynes generated from aryl triflates in flow, using tetrabutylammonium fluoride (TBAF) as a fluoride source.^12^ Heretsch, Christmann, and co-workers later reported the use of aryl triflates to generate arynes in flow toward the synthesis of quinolones.^13^ Tetrabutylammonium difluorotriphenylsilicate (TBAT) was chosen as a soluble fluoride source. Initial optimization of the insertion of arynes into unsymmetric imides was carried out in batch and gave the desired isomer in a modest 35% yield at 80 °C. This was attributed to thermal decomposition of the starting imide; however, elevated temperatures were needed for satisfactory conversion to the desired product. By transferring the reaction to flow, this decomposition could be suppressed through using shorter reaction times and lower temperatures. Following optimization, **3** could be isolated in a much improved 52% yield at 65 °C. Using a flow setup consisting of two syringe pumps and a heated 4 mL reactor, a stream of aryl triflate and imide was combined with a stream of TBAT and passed through the coil reactor with a residence time of 4 min (Scheme 1).

**Scheme 2:**
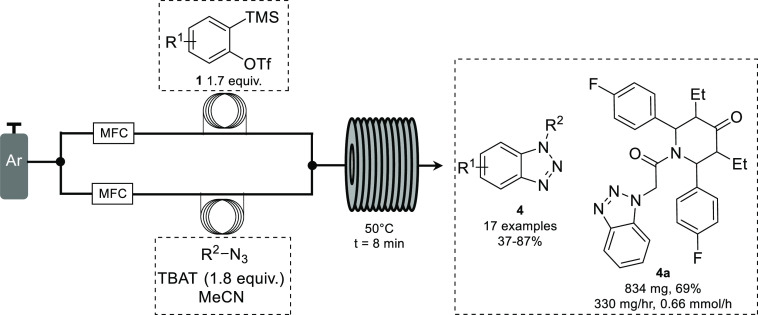
Synthesis of Benzotriazoles in Continuous Flow Mode

The reaction of benzyne with azides is well documented.^14−16^ However, heating of organic azides and highly reactive benzyne intermediates raises significant safety concerns when aiming to scale this reaction. Recently, the Heretsch group disclosed the use of arynes, generated in a similar manner, toward the synthesis of benzotriazoles in flow.^17^ Using argon gas to drive reagents, the aryne precursor was combined with a stream of organic azide and fluoride source and passed through a coil reactor with a residence time of 8 min (Scheme 2). A range of benzotriazoles (**4**) were obtained in good yields when the reaction was carried out at 50 °C. Various substrates were tolerated including ferrocenyl azides, which are typically explosive when heated. Importantly, the scalability of the reaction was demonstrated by synthesizing **4a** on a gram scale with a productivity of 0.33 g/h (0.66 mmol/h). The scale-up was realized using sealed sample flasks containing substrate solutions, from which the solutions were driven through the reactor using the stream of argon gas.

**Scheme 3:**
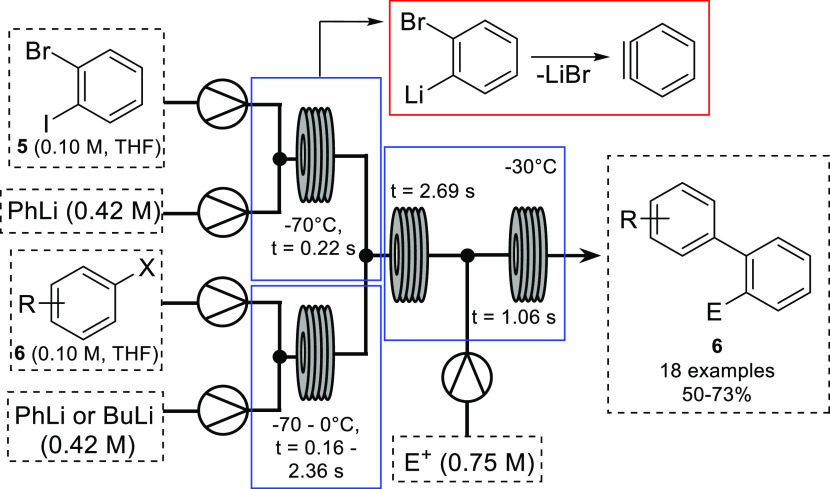
Synthesis of Biaryl Compounds from Benzyne

Arynes can also be generated via metal–halogen exchange/elimination processes involving dihaloarenes. This, however, presents various challenges due to the low temperature and accurate reagent dosing required. There are multiple examples of metal–halogen exchange reactions in continuous flow benefiting from the improved parameter control.^7,18^ In 2014, the Yoshida group disclosed the lithiation/elimination of 1,2-dihalobenzenes to generate benzynes in continuous flow.^19^ The reaction of benzyne with aryllithiums can be used to generate biaryllithiums, which can then further react with an electrophile. While useful, biaryllithiums are highly reactive and their use in batch is near impossible due to their short lifetimes. The Yoshida group exploited the benefits of flash chemistry^20,21^ to first generate the required benzyne and subsequent biaryllithium intermediates at low temperature, followed by reaction with a range of electrophiles at −30 °C to provide the desired biaryl products **5** in yields of 50–73% (Scheme 3). Using subsecond residence times, the high reactivity of the biaryllithium intermediate was exploited.

**Scheme 4:**
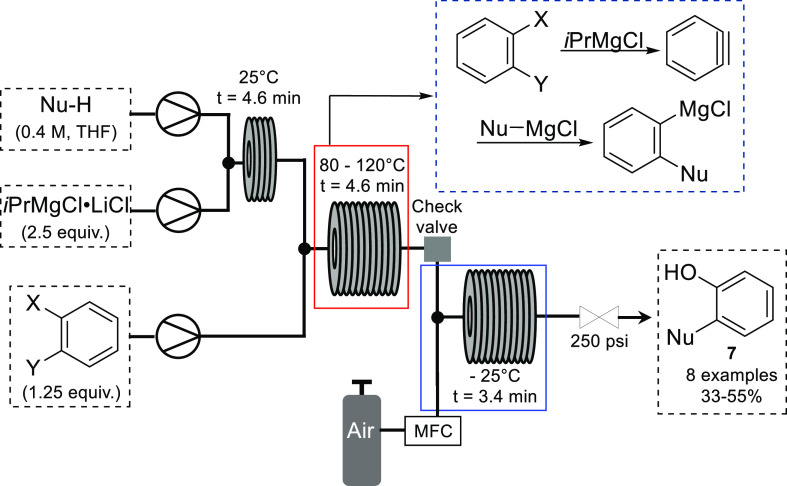
Continuous Flow Synthesis of Phenols from Benzyne

Similarly, He and Jamison reported the generation of Grignard reagents via the addition of an organo-magnesium species to benzyne in flow.^22^ Reacting 1,2-dihalobenzenes with an organo-magnesium species, followed by *in situ* oxidation using compressed air, yielded phenol **7** in moderate yield. The flow setup adopted a telescoped approach with initial generation of the organo-magnesium species taking place at room temperature, followed by benzyne generation at 80–120 °C and subsequent oxidation at −25 °C (Scheme 4). The temperature required for benzyne generation depended on the susceptibility of the precursor to metal–halogen exchange, with 1,2-dibromobenzene being sufficiently reactive at 80 °C.

**Scheme 5:**
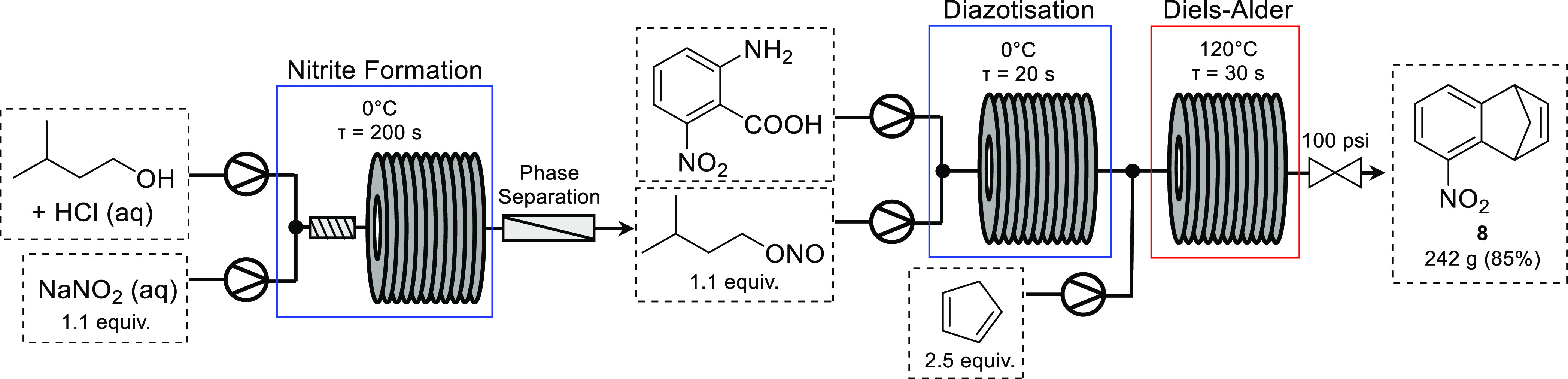
Telescoped Synthesis of 5-Nitro-1,4-dihydro-1,4-methanonaphthalene

Diazotization of 2-aminobenzoic acids can be utilized to generate arynes, following expulsion of CO_2_ and N_2._^23^ However, there are several hazards associated with this reaction, particularly when scaling up. Diazonium salts are potentially explosive; in addition to the high reactivity of the resulting benzyne intermediate and the formation of gas, this reaction can be challenging to safely perform on scale. Several examples of the continuous flow diazotization of anilines have been reported.^1^ In 2012, the Ley group reported the continuous flow diazotization of anthranilic acid and subsequent generation of benzyne followed by Diels–Alder reaction with cyclopentadiene.^24^ The use of in-line mass spectrometry allowed for the monitoring of the reaction and highlighted potentially hazardous side products generated during benzyne formation. Recently, Tan and co-workers reported the use of 2-nitrobenzyne toward the synthesis of 5-nitro-1,4-dihydro-1,4-methanonaphthalene, a useful drug intermediate.^25^ Using a telescoped approach, isoamyl nitrite was synthesized from isoamyl alcohol and subsequently reacted with 6-nitro-2-aminobenzoic acid to form the corresponding diazonium salt. Thermal decomposition of this diazonium salt in the presence of cyclopentadiene provided product **8** in 85% yield (Scheme 5). The reaction was scaled to give 242 g of product (∼2.4 g/min), offering significant improvements when compared to batch.

**Scheme 6:**
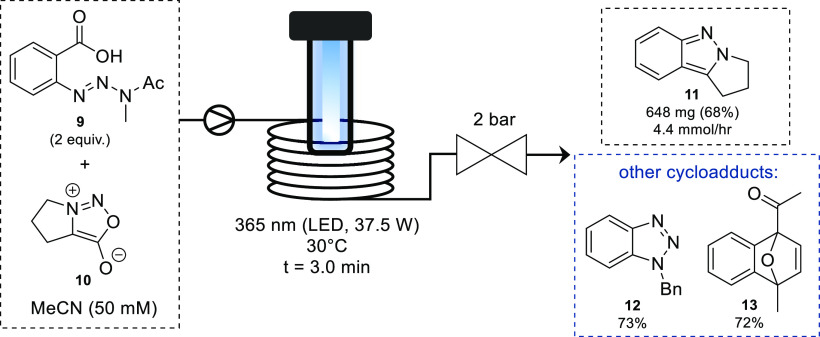
Photochemical Generation of Benzyne

A similar method for the formation of benzyne involves the photochemical decomposition of benzoic acid triazines, which produces benzyne with the liberation of acetamide, N_2_, and CO_2_.^26^ Recently, Bracken and Baumann reported the continuous photochemical generation of benzyne exploiting the favorable properties of flow reactors for photochemical processes.^27^ Through careful temperature control and selection of light source, benzyne cycloadducts could successfully be isolated in good yields. Using a 365 nm LED and backpressure regulator (BPR), the photochemical process was demonstrated using a range of azides, dienes, and sydnones as reaction partners (Scheme 6). The scalability of the process was highlighted by synthesizing 2*H*-indazole **11** with a throughput of 4.4 mmol/h, which would be difficult to achieve using a comparable batch setup.

## 2 Use of Carbenes in Continuous Flow

Carbenes and carbenoids have found wide utility in organic synthesis, particularly for cyclopropanation and C–H insertion reactions.^28−31^ Carbenes are typically generated through either photochemical decomposition or reaction of a precursor with a transition metal to form a carbenoid. Free carbenes are generally more reactive than the corresponding carbenoids and require precise control to exploit.

Carrying out reactions in continuous flow allows for the heating of solvents beyond their boiling point at atmospheric pressure through the application of back-pressure. This allows reactions to be carried out at higher temperatures than would be possible in batch (without the use of pressurized reaction vessels), which can provide shorter reaction times and higher productivity.

**Scheme 7:**
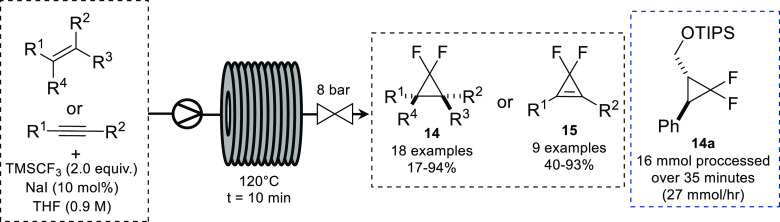
Generation of Difluorocarbene in Flow

In 2016, the Charette group exploited this characteristic to develop a scalable platform for the synthesis of difluorocyclopropanes and -propenes.^32^ The use of TMSCF_3_ as a difluorocarbene source had previously been reported in batch^33^ but required reaction times of 2 h and, in the case of difluorocyclopropenes, heating THF to 110 °C. The resulting flow setup consisted of a Vapourtec R-series reactor equipped with a 10 mL coil reactor heated to 120 °C and an 8 bar BPR. The desired difluorocyclopropane/propene (**14/15**) was synthesized in 10 min from the corresponding alkene/alkyne. To demonstrate the scalability of the method, cyclopropane **14a** was synthesized on a multigram scale from the corresponding alkene (Scheme 7).

**Scheme 8:**
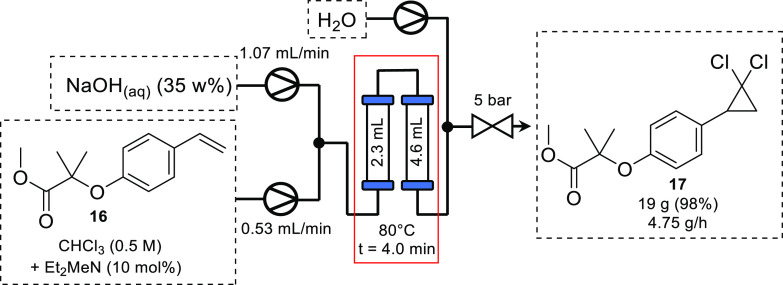
Continuous Flow Synthesis of Ciprofibrate Methyl Ester **17**

Another method for the generation of dihalocarbenes is the reaction of chloroform in the presence of NaOH and, typically, a phase-transfer catalyst (PTC). This method is often used for the synthesis of dichlorocyclopropanes; however, it can be inefficient due to poor mixing of the biphasic mixture, causing the generation of insoluble side products. Carrying out reactions in flow can improve mixing profiles due to the improved mass transfer observed in micro- and meso-reactors. This mixing efficiency can drop over the course of a biphasic reaction as the residence time increases, due to phase separation occurring. Recently, the Kappe group developed a continuous flow platform for the synthesis of dichlorocyclopropanes utilizing this aqueous/chloroform system with dimethylethylamine as a PTC.^34^ Using standard T-piece mixers, a segmented flow was achieved; however, it was observed that the conversion of cyclohexene to the corresponding dichlorocyclopropene plateaued as residence time increased due to phase separation occurring at longer residence times (50% conversion after 5 min, 56% after 10 min). Through process optimization, a consistent emulsion could be achieved by using a packed bed reactor containing PTFE beads. This improved mixing and resulted in full conversion of cyclohexene to the desired cyclopropane, affording the product in 97% yield (80 °C, 4 min residence time). The scalability of the system was then demonstrated by synthesizing Ciprofibrate methyl ester **17** from the alkene **16** on a decagram scale with high yield and throughput (Scheme 8).

In contrast to the discussed carbenes which are generated under relatively harsh conditions, metal carbenoids can provide access to carbene-like reactivity at low temperatures. Metal carbenoids typically consist of a tetracoordinated carbon atom bound to a metal (M) and a leaving group (X).^35^ The carbene species can then be transferred to the reactive partner through the loss of MX. A well-reported example of this reactivity is the Simmons–Smith reaction.^36^

**Scheme 9:**
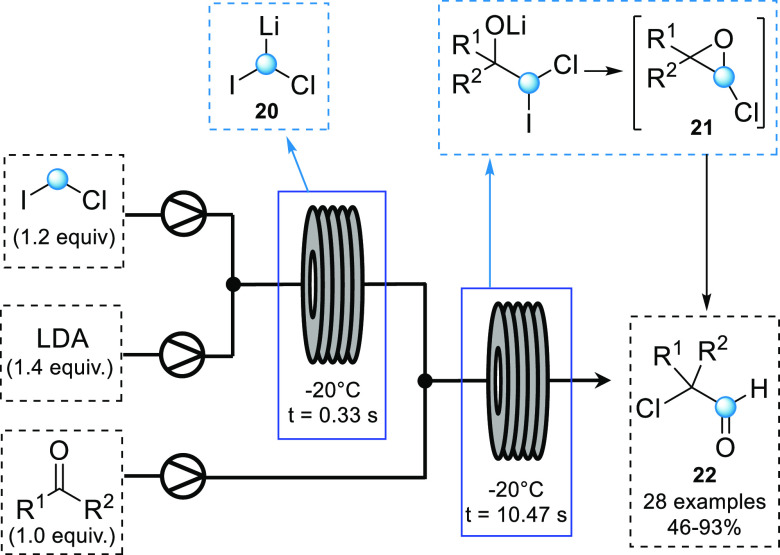
Continuous Flow Generation of Lithium–Carbenoid

In 2019, the Kappe group reported the generation of lithium–carbenoid from bromomethyl lithium and its subsequent reaction with ketones to form epoxides in continuous flow.^37^ More recently, the Luisi group disclosed the use of chloroiodomethyllithium carbenoid in flow for the direct synthesis of α-chloroaldehydes from the corresponding ketones.^38^ The desired carbenoid **20** could be formed through reaction of chloroiodomethane with LDA. Using a one-pot “internal quenching” batch procedure with benzophenone at −78 °C, the desired α-chloroaldehyde was isolated in 55% yield. However, preforming the carbenoid prior to adding in the ketone reaction partner after 1 min (external quenching) resulted in a significantly decreased yield of 18%. To determine the lifetime of the lithium carbenoid, the reactive species was quenched using CD_3_OD at various temperatures and reaction times in flow. A residence time of 330 ms at −20 °C was optimal for maximizing the generation of the carbenoid (94% recovery). This lifetime could be further increased to 9.4 s by decreasing the temperature to −78 °C. A range of α-chloroaldehydes were synthesized with a total residence time of 10.8 s (Scheme 9). A significant increase in yield was observed when compared to the external quench batch method. It is worth noting that some substrates required subsequent acidic treatment to facilitate the Meinwald-type rearrangement of chloroepoxide **21** to the desired aldehyde.

Rhodium metal carbenes generated from diazo compounds are some of the most used carbene sources.^39,40^ In batch processes, this typically involves the use of soluble dirhodium catalysts which are difficult to recover. Due to the significant expense of the catalyst, maximizing the turnover number and recoverability of these complexes is crucial. The use of immobilized reagents in continuous flow mode is a commonly utilized method for increasing the efficiency of such reagents.^41,42^ As the substrates pass through the reactor, they experience a local excess of catalyst/reagent which typically leads to shorter reaction times. Additionally, the reusability of these reactors proves advantageous.

In 2018, Yoo, Rackl, and co-workers disclosed the development of a flow reactor which takes advantage of these characteristics.^43^ By combining their previously reported method^44^ for the flow synthesis of diazo compounds from the corresponding hydrazones, using a polymer-supported *N*-iodo *p*-toluenesulfonamide (PS-SO_2_NIK) resin, with an immobilized dirhodium hollow-fiber reactor, a telescoped system for C–H functionalization reactions was developed. The dirhodium catalyst was grafted to polymeric fibers embedded with silica particles, which were subsequently wrapped with PTFE to provide a nonpermeable tube reactor. The continuous flow setup consisted of a PS-NIK resin packed column, the output of which was mixed with the desired coupling partner in a T-piece mixer and passed through a column containing Na_2_S_2_O_3_ and molecular sieves to remove impurities from the diazo forming reaction. This second column was connected to the Rh-immobilized reactor. Using a chiral Rh catalyst, a range of cyclopropanation and C–H insertion reactions were carried out, with yields comparable to previous batch results. Investigation of the recyclability of the catalyst found that after 10 uses yields dropped slightly from 74% to 65% for the benzylic C–H insertion of 4-methoxytoluene, with enantioselectivity remaining relatively constant at 89–86% ee.

More recently, Masuda and co-workers reported the use of Rh nanoparticles supported on nitrogen-doped carbon as a solid ligand in continuous flow.^45^ The N–H insertion of diazoesters to synthesize chiral amines was investigated with throughputs of up to 4 g/day reported. The Maguire group have also reported the telescoped synthesis of diazo compounds and subsequent S–H insertion using a Rh catalyst.^46^

An alternative method for the generation of carbenes is the photochemical decomposition of diazoalkanes.^47^ Various diazo esters absorb light in the visible region to generate the corresponding carbene following loss of nitrogen. Carrying out photochemical reactions in flow offers various advantages, primarily stemming from shorter path lengths allowing for more intense and uniform irradiation of solutions.^2^

Important contributions from the Ley group demonstrate the use of oxadiazolines as precursors of nonstabilized diazo species that were converted to various valuable products under continuous photochemical conditions.^48−50^ More recently, the Koenigs group reported the generation of carbenes from aryl diazoesters using blue light (470 nm) in flow.^51^ The cyclopropenation of alkynes and the Doyle–Kirmse rearrangement of sulfides were studied. While the reactions proceeded smoothly in batch with the corresponding carbene successfully generated and trapped by a wide range of substrates, reaction times were lengthy (16 h) and provided the products in low throughput. The cyclopropenation of phenylacetylene with methyl 2-diazo-2-phenylacetate was transferred to flow. Using a glass microreactor (1.0 mL) which was irradiated using the same light source (3 W LED, 470 nm) as in batch, the reaction time could be reduced to 5 h, albeit with no improvement in throughput. Increasing the power of the light source to 24 W, despite a drop in yield (99% to 72%), led to an increase in productivity through a reduction of reaction time to 0.5 h. Using a similar setup, the Koenigs group also reported the continuous flow cyclopropanation of styrene using diazoesters.^52^

**Scheme 10:**
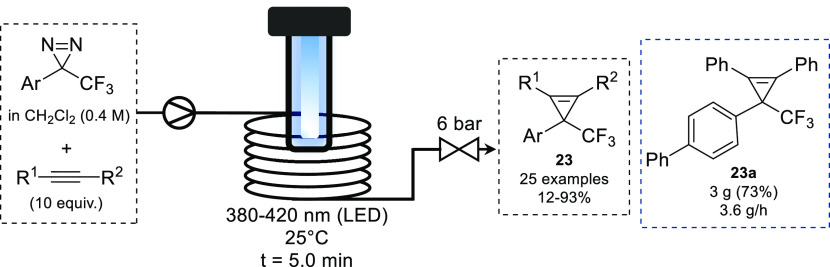
Photochemical Cyclopropenation of Acetylenes

Similar reactivity can also be achieved via the photochemical decomposition of diazirines.^53^ In 2021, the Ollevier group reported the use of diazirines to access trifluoromethyl cyclopropenes.^54^ A range of light sources and diazirine precursors were investigated for the cyclopropenation of diphenylactylene. As expected, the wavelength of the light source was dependent on the structure and absorbance profile of the starting diazirine, with some diazirines being active at 420 nm and others requiring more energetic light (405 or 380 nm). The optimal reaction conditions were found to be a residence time of 5 min at 25 °C. Using these conditions, a range of cyclopropenes were synthesized (Scheme 10). A comparison of batch and flow found that significantly longer reaction times (24 h) were required for partial conversion in addition to lower yields in batch. The cyclopropenation of diphenylacetylene to provide **23a** was carried out on 10 mmol scale, with the product isolated on multigram scale after 50 min, albeit at a lower yield than observed at small scale (73% vs 93%).

**Scheme 11:**
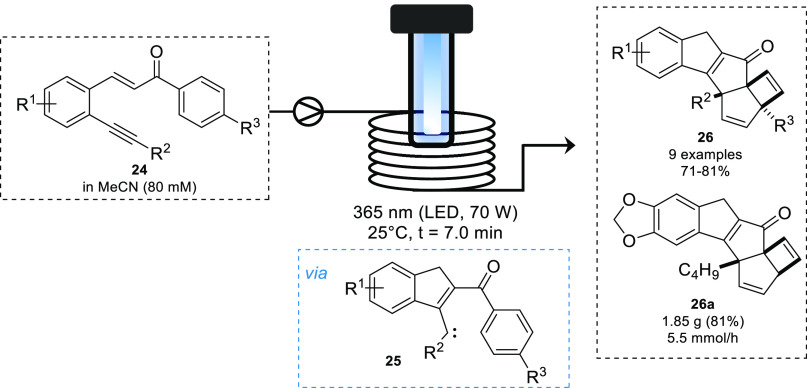
Unusual Photochemical Rearrangement of Chalcones via Carbene Intermediates

In addition to being used as stoichiometric reagent, carbenes can act as key intermediates during intramolecular reactions, as observed by the Baumann group in the photochemical cyclization of chalcones.^55^ Building on previous work investigating the intramolecular cyclization of chalcones to synthesize quinolines,^56^ a recent study utilized chalcones bearing an alkyne group. Irradiation of the alkyne bearing chalcone **24** led to the complex polycyclic product **26a**. Following a short irradiation of 7 min using a tunable high powered LED source (365 nm), this product could be isolated in up to 81% yield. Exploiting the advantages associated with photochemical flow processes,^2,3^ the reaction was scaled out to 1 h to provide 1.85 g of **26a** (5.5 mmol/h), which was then used to determine the structure of this unexpected cascade product. Following computational investigations into the reaction mechanism it was confirmed that the product is formed *via* a key carbene intermediate **25**, followed by subsequent norcaradiene formation and electrocyclization reactions (Scheme 11).

## 3 Nitrenes in Continuous Flow

Nitrenes are key intermediates in the formation of C–N bonds and display reactivity similar to that of their carbon-containing equivalents, carbenes.^28^ The development of nitrene reactions has evolved significantly in recent decades to now include the aminohydroxylation of alkenes, cycloadditions, and various cascade reactions.^57^ A commonly exploited reaction of nitrenes is the aziridination of alkenes. Aziridines serve as useful reactive intermediates to access other amino containing compounds due to their propensity to ring open in the presence of nucleophiles. However, aziridines are inherently toxic due to their high potency as alkylating agents; thus, exposure when working with these compounds must be limited.

**Scheme 12:**
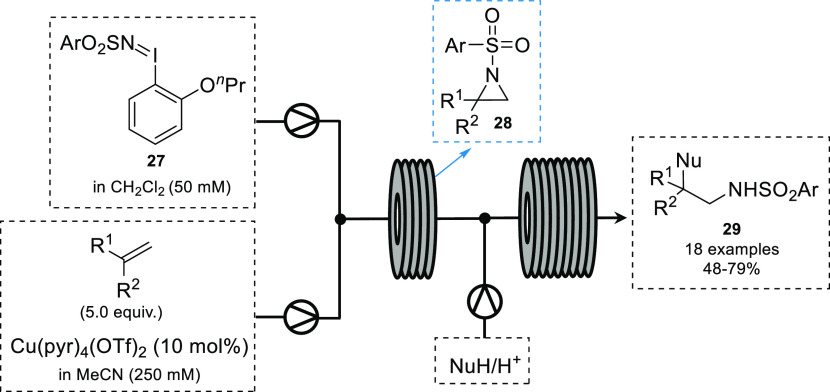
Telescoped Synthesis of Amino Compounds **29** via Aziridine **28**

In 2016, the Shipman group reported the telescoped continuous flow synthesis of aziridines and their subsequent ring opening.^58^ Arylsulfonylimino phenyliodanes can be used as nitrene precursors; however, some commonly used derivatives such as those based on tosyl and nosyl systems (PhI = NTs and PhI = NNs) are insoluble in organic solvents, making them unsuitable for use in flow. Through modification of the iminoiodane structure improved solubility can be achieved.^59^ Using iminoiodane **27** as a nitrene precursor in the presence of tetrakis(pyridine)copper(II) triflate (10 mol %) and alkene (10 equiv), the corresponding aziridne could be synthesized in 10 min using a chip reactor (volume = 4.5 mL). As some aziridines proved to be unstable, a telescoped system was designed where dosing of a nucleophile through a second chip reactor would provide the desired amino compound (Scheme 12).

While various copper-catalyzed nitrene forming reactions are reported, a number require the use of insoluble catalysts which are incompatible with continuous flow systems. To overcome this issue, a group from Novartis recently reported the use of a copper coil reactor as a replacement for insoluble Cu_2_O for the cyclization of aryl azides via a nitrene intermediate.^60^ While this reaction worked efficiently in batch, a safe and scalable reaction was required for the scaled synthesis of a 2*H*-indazole intermediate. In flow mode, contact with the potentially explosive azide was avoided. Additionally, the setup provided 217 g of desired product in a high throughput of 8.4 g/h using a telescoped process starting from the corresponding aminoaldehyde.

Similarly, the aziridination of olefins can be carried out in the presence of an alternative metal catalyst such as ruthenium, which was reported in continuous flow by Rossi et al.^61^ The simple setup consisted of a small coil reactor (500 μL) and a syringe pump to dose the reagents. Using the desired olefin as the reaction solvent and a Ru–porphyrin catalyst, the corresponding aziridine could be isolated in moderate to high yields (55–98%) in 30 min when heated to 120 °C. Carrying out the reaction in flow did not offer any improvements in yield or productivity when compared to batch mode; however, it does avoid safety issues associated with the handling of organic azides.

**Scheme 13:**
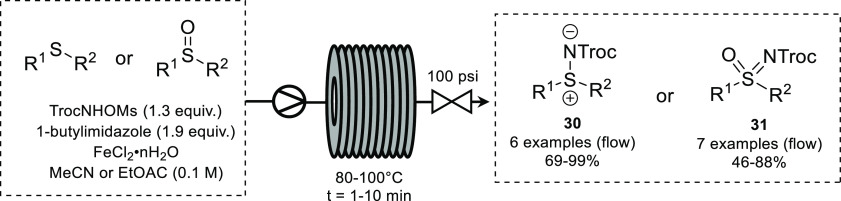
Continuous-Flow Amination of Sulfides and Sulfoxides

Nitrenes can also be used for the amination of sulfides as described by the Lebel group.^62^ The reaction of 2,2,2-trichloroethyl-*N*-mesyloxycarbamate (TrocNHOMs) with sulfides or sulfoxides in the presence of base and FeCl_2_ provided the corresponding sulfimines and sulfoximines in moderate to good yields. Use of 1-butylimidazole as a base to form an ionic liquid byproduct in place of an insoluble salt allowed the reaction to be transferred from batch to flow. While a range of sulfimines and sulfoximines could be synthesized under batch conditions in 15 and 60 min, respectively, transferring the reaction to flow reduced these times to 1–10 min (Scheme 13). Improvements in yields were observed in some substrates, and access to compounds which were not isolable in batch was possible.

**Scheme 14:**
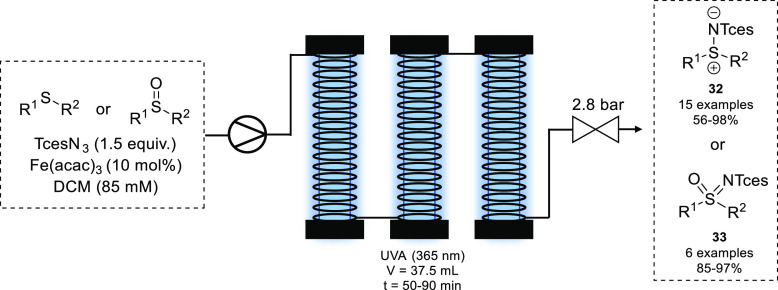
Photochemical Amination of Sulfides and Sulfoxides via Iron Nitrenoid

In 2016, the Lebel group also achieved the amination of sulfides and sulfoxides using organic azides as a nitrene source.^63^ They found that in the presence of Fe(acac)_3_ and UV-A light (365 nm), trichloroethoxysulfonyl azide (TcesN_3_) could be used for the amination of sulfides and sulfoxides. Using a bespoke photochemical reactor consisting of PFA tubing wrapped around 8 W UV-A tubes, the corresponding sulfimines (**32**) and sulfoximines (**33**) were isolated in 50–90 min (Scheme 14). Use of alternative azides such as TsN_3_ or TrocN_3_ did not result in product formation.

**Scheme 15:**
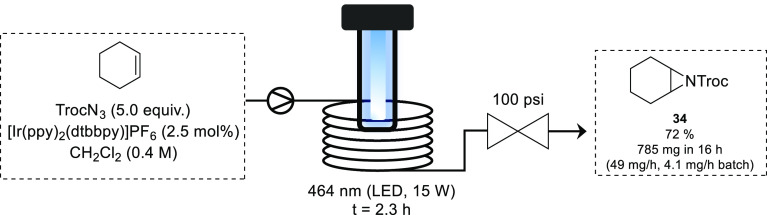
Photochemical Aziridination Using Triplet Nitrene

While aziridination reactions are commonly carried out using nitrenoids, the use of free nitrenes is challenging due to their high reactivity and instability. Like carbenes, nitrenes can exist in the triplet or singlet state, with different reactivity associated with either state. Triplet carbethoxynitrenes react selectively with alkenes to form aziridines, whereas their singlet counterpart undergoes both amination and aziridination.^64^ Using a triplet sensitizer, the Yoon group found that selective photochemical aziridination could be achieved using TrocN_3_.^65^ Using blue light (464 nm) and an Ir-photocatalyst a range of aziridines were synthesized in batch in 20 h (0.4 mmol scale). Changing to a more energetic light source (315 nm) resulted in a competing allylic amination process. By transferring to flow, the reaction time could be reduced to 2.3 h to provide **34** in similar yield to batch. Scaling out of the process yielded 785 mg of product in 16 h equating to a more than 10-fold increase in productivity compared to batch mode (Scheme 15).

In some cases, singlet nitrene generation is favorable, as reported by Seeberger and co-workers in their preparation of 3*H*-azepinones.^66^ The photolysis of aryl azides in the presence of water was exploited to access a range of 3*H*-azepinones. The reaction proceeds via the ring expansion of a 2*H*-azirine formed by a singlet nitrene, and the corresponding triplet nitrene underwent dimerization to give an undesired diazo compound. The flow setup consisted of a FEP coil reactor surrounding a medium-pressure Hg lamp, which afforded a range of 3*H*-azepinones in moderate to good yields with a residence time of 30 min. While yields were comparable to batch, carrying out the reaction in flow provides a more scalable process.

## 4 Conclusion

The use of miniaturized continuous flow systems allows for efficient exploitation of highly reactive intermediates. Through the combination of high mass and heat transfer, in addition to improved efficiencies for photochemical reactions, flow chemistry has provided access to previously undescribed reactivity. This presents the opportunity to access chemical space which was previously unavailable and to accelerate the discovery of novel reactions. While some of the areas described herein remain underdeveloped, particularly the use of nitrenes, the development of flow methodologies may accelerate their widespread use and drive new innovations in the field.
